# Allogeneic HSCT for Symptomatic Female X-linked Chronic Granulomatous Disease Carriers

**DOI:** 10.1007/s10875-023-01570-z

**Published:** 2023-08-24

**Authors:** Christo Tsilifis, Tuulia Torppa, Eleri J. Williams, Michael H. Albert, Fabian Hauck, Elena Soncini, Elizabeth Kang, Harry Malech, Catharina Schuetz, Horst von Bernuth, Mary A. Slatter, Andrew R. Gennery

**Affiliations:** 1 Paediatric Haematopoietic Stem Cell Transplant Unit, Great North Children’s Hospital, Ward 3, Newcastle Upon Tyne, NE1 4LP UK; 2 Translational and Clinical Research Institute, Newcastle University, Newcastle Upon Tyne, UK; 3 School of Medicine, Faculty of Medical Sciences, Newcastle University, Newcastle Upon Tyne, UK; 4 Department of Pediatrics, Dr. Von Hauner Children’s Hospital, University Hospital, LMU, Munich, Germany; 5 Paediatric Haematopoietic Stem Cell Transplant Unit, Children’s Hospital ASST Spedali Civili, Brescia, Italy; 6 Laboratory of Clinical Immunology and Microbiology, National Institute of Allergy and Infectious Diseases, National Institutes of Health, Bethesda, MD USA; 7 Department of Pediatrics, Medizinische Fakultät Carl Gustav Carus, Technische Universität Dresden, Dresden, Germany; 8 Department of Pediatric Respiratory Medicine, Immunology, and Critical Care Medicine, Charité-Universitätsmedizin Berlin, Berlin, Germany; 9 Department of Immunology, Labor Berlin Charité-Vivantes, Berlin, Germany; 10 Berlin Institute of Health at Charité – Universitätsmedizin Berlin, Berlin, Germany; 11 Berlin-Brandenburg Center for Regenerative Therapies (BCRT), Charité - Universitätsmedizin Berlin, Corporate member of Freie Universität Berlin, Humboldt-Universität Zu Berlin, and Berlin Institute of Health (BIH), Berlin, Germany

**Keywords:** Allogeneic HSCT, Chronic granulomatous disease, X-linked carrier, Lyonization

## Abstract

X-linked chronic granulomatous disease (XL-CGD) is an inherited disorder of superoxide production, causing failure to generate the oxidative burst in phagocytes. It is characterized by invasive bacterial and fungal infections, inflammation, and chronic autoimmune disease. While XL-CGD carriers were previously assumed to be healthy, a range of clinical manifestations with significant morbidity have recently been described in a subgroup of carriers with impaired neutrophil oxidative burst due to skewed lyonization. Allogeneic hematopoietic stem cell transplantation (HSCT) is the standard curative treatment for CGD but has rarely been reported in individual symptomatic carriers to date. We undertook a retrospective international survey of outcome of HSCT for symptomatic XL-CGD carriers. Seven symptomatic female XL-CGD carriers aged 1–56 years underwent HSCT in four centers, indicated for severe and recurrent infection, colitis, and autoimmunity. Two patients died from transplant-related complications, following donor engraftment and restoration of oxidative burst. All surviving patients demonstrated resolution of their neutrophil oxidative burst defect with concordant reduction in infection and inflammatory symptoms and freedom from further immunosuppressive therapy. In conclusion, allogeneic HSCT may cure the phagocyte defect in symptomatic XL-CGD carriers and improve their recurrent and disabling infective and inflammatory symptoms but risks transplant-related complications.

## Introduction

Chronic granulomatous disease (CGD) is an inherited disorder of phagocytes characterized by recurrent and severe bacterial and fungal infections, and inflammatory and autoimmune manifestations including colitis. It is caused by a failure of oxidative burst formation, due to defects in the nicotinamide dinucleotide phosphate (NADPH) complex required for generation of superoxides. CGD arises from pathogenic mutations in genes encoding subunits of the NADPH complex: *CYBA*, *CYBC1*, *NCF1*, and *NCF2*, inherited in an autosomal recessive fashion, or *CYBB*, which has X-linked inheritance [1]. Biallelic mutations in *NCF4* underlie a related, but clinically distinct and milder form of CGD [2]. While the prevalence of which gene is impacted varies geographically, the most common mutations causing CGD in outbred populations are in *CYBB*, manifesting as X-linked CGD (XL-CGD) [3].

Female XL-CGD carriers have a dual population of functioning and non-functioning neutrophils due to random X-chromosome inactivation, and typically are not susceptible to classical CGD-related infections due to sufficient residual oxidative burst from their functioning neutrophil population. However, this cohort carries an increased susceptibility to autoimmune manifestations such as lupus-like disease and may suffer from delayed wound healing. In addition, it is increasingly recognized that a subgroup of XL-CGD carriers with skewed inactivation of the wild-type allele resulting in low oxidative burst activity due to lyonization present with a range of clinical manifestations consistent with classical CGD [4]. In these symptomatic XL-CGD carriers, risk of infection and chronic gastrointestinal inflammatory symptoms like colitis may be predicted by low dihydrorhodoamine-123 oxidation (%DHR +). The autoinflammatory tendency seen in these patients is postulated to originate from deficient efferocytosis, through prolonged exposure to damage-associated molecular patterns (DAMPs) leading to immune hyperactivation [5]. Defective efferocytosis has been implicated in the pathogenesis of impaired wound healing and systemic lupus erythematosus, of which several features, including auto-antibody formation, may occur in XL-CGD carriers [6, 7]. Furthermore, NADPH-deficient macrophages promote effector T-lymphocyte activation and expansion, with enhanced secretion of pro-inflammatory cytokines such as IL-17 and IL-18, which may contribute further to the inflammatory milieu [8, 9]*.* XL-CGD patients also demonstrate an altered B-lymphocyte compartment [10], together suggesting that while CGD affects innate cells, the adaptive arm of immunity is also impacted. Identification of which immune mechanisms lead to different inflammatory manifestations in CGD and XL-CGD carriers has yet to be explored. Infection, colitis, and other autoimmune manifestations may lead to repeated hospitalization, fatigue, and poor quality of life [4, 11–16]. Neutrophil function in carriers may be dynamic, with progression of skewing of lyonization with increasing age. Therefore, some patients without a positive family history may present later in life [13].

Allogeneic hematopoietic stem cell transplantation (alloHSCT) has become the standard curative treatment for patients with CGD, with excellent outcome in respect of mortality, reduction in hospitalization, resolution of chronic infection and inflammatory symptoms, and improvement in quality of life [17–22], particularly considering poor outcomes for un-transplanted adults who accumulate organ morbidity as they age. For symptomatic XL-CGD carriers, the role of alloHSCT is less well-described, with two patients previously reported [16, 23].

To better understand the impact and limitations of alloHSCT in this patient group, we describe the clinical and immunological outcomes for seven symptomatic XL-CGD carriers with severe disease manifestations treated with alloHSCT, including updated follow-up for the two previously reported patients.

## Methods

Clinical, laboratory, and immunological data were collected retrospectively from written and electronic medical records of seven symptomatic XL-CGD carrier patients who had undergone alloHSCT across four sites, for which patients had given prior written consent. Patients were identified through contact with inborn errors of immunity transplant societies in Europe, North America, and Australia, as well as the European Bone Marrow Transplant and Stem Cell Transplantation for Immunodeficiencies in Europe registries. Statistical analysis was not feasible due to the limited sample size. Patients treated at the National Institutes of Health, Bethesda, MD (P4 and P5) were treated under protocol NIH 15-I-0008.

## Case Reports

Seven unrelated patients were included: all were female XL-CGD carriers, six with heterozygous pathogenic mutations or deletions in *CYBB*, and one with a large deletion incorporating *CYBB*. All patients had clinical features consistent with CGD or with symptomatic XL-CGD carrier status. Age at HSCT ranged between 1 and 56 years and survivors were followed for 1.5–7.5 years post-HSCT. Clinical details, genetics, and pre-HSCT %DHR + are summarized in Table 1, while transplant data and outcome are summarized in Table 2.

**Table 1:** Clinical features, genetics, and pre-HSCT %DHR + . Key: ECMO, extracorporeal membrane oxygenation

Patient	Reason for %DHR + testing	Age at diagnosis (years)	Clinical features and age of onset	CYBB mutation	%DHR +	Indication for HSCT
P1	Family history	Birth	Discoid rash, 1 year Polyarthralgia requiring wheelchair, 1 year Recurrent pneumonia and lymphadenitis, 4 years Colitis, 6 years	Deletion of exons 6–13	25%	Lupus-like disease unresponsive to multiple lines of immunosuppression; colitis
P2	Clinical presentation	16	Recurrent folliculitis, 12 years *S. aureus* liver abscess, 16 years	Large deletion (Xp.21.1p.11.4)	10%	Life-threatening infection
P3	Clinical presentation	17	Severe colitis, 9 years	c.925G > A	30%	Colitis unresponsive to therapy; matched donor available
P4	Clinical presentation	49	Nocardiosis requiring ECMO, 49 years Recurrent pneumonia, 51 years Colitis Discoid lupus and Raynaud’s Hypothyroidism	c.1662dupT	6.7%	Life-threatening infection with multi-organ autoimmunity/inflammation
P5	Clinical presentation	2	Nocardiosis with lung resection, 2 years Lupus Colitis with colo-vaginal and colo-labial fistulae	c.1548G > A	3.5%	Fistulizing colitis refractory to immunosuppression; infections
P6	Clinical presentation	6	*Burkholderia gladioli* abscess Pneumonia Discoid rash Hypogammaglobulinaemia	c.1151 + 2 T > C	15%	Severe and recurrent infection
P7	Family history	Birth	Bilateral pneumonia Colitis	c.483 + 1G > A	1.2%	Infection and colitis, low %DHR + , family history

**Table 2:** Transplantation details, outcome, and clinical status post-HSCT. Key: AIHA, autoimmune hemolytic anemia; *AUC*, area under the curve; *BM*, bone marrow; *CsA*, ciclosporin A; *MMF*, mycophenolate mofetil; *MSD*, matched sibling donor; *MUD*, matched unrelated donor; *PBSC*, peripheral blood stem cell; *PTCy*, post-transplant cyclophosphamide

Patient	Age at HSCT (years)	Stem cell source and HLA match	Conditioning regimen Serotherapy	GvHD prophylaxis	GvHD and treatment	Follow-up duration (years)	Latest donor chimerism	Latest %DHR +	Outcome	Post-HSCT infection	Post-HSCT inflammation or autoimmunity
P1	15	10/10 MUD-PBSC	Treosulfan 42 g/m^2^ Fludarabine 150 mg/m^2^ Thiotepa 10 mg/kg Alemtuzumab 1 mg/kg	CsA/MMF	-	4	100%	100%	Survival	None	AIHA — resolved Rash and arthralgia in remission
P2	18	10/10 MUD-PBSC	Treosulfan 42 g/m^2^ Fludarabine 150 mg/m^2^ Thiotepa 10 mg/kg Alemtuzumab 1 mg/kg	CsA/MMF	-	-	100%	100%	Death D + 30 — toxic leukoencephalopathy from conditioning agents	-	-
P3	20	10/10 MUD-BM	Busulfan (AUC: 44 mg/L*h) Fludarabine 180 mg/m^2^ Alemtuzumab 0.6 mg/kg	CsA/MMF	Grade I skin — topical steroids	8	100%	100%	Survival	None	Clinical and histological remission from colitis
P4	56	10/10 MSD-PBSC	Busulfan (AUC: 49 mg/L*h) Alemtuzumab 1 mg/kg	PTCy Sirolimus	Grade I skin — topical steroids	3	60%	60%	Survival	None	Clinical remission from colitis and lupus Hypothyroidism continues
P5	22	Haplo-PBSC	Busulfan (AUC: 49 mg/L*h) Fludarabine 150 mg/m^2^ Cyclophosphamide 29 mg/kg	PTCy Sirolimus	Grade 3 GI/liver — corticosteroids, infliximab	-	100%	98%	Death D + 100 — multiorgan failure from disseminated adenovirus and GvHD	-	-
P6	7	10/10 MSD-BM	Busulfan (AUC: 91 mg/L*h) Fludarabine 160 mg/m^2^ Alemtuzumab 0.6 mg/kg	CsA	-	2	100%	100%	Survival	None	None
P7	1	TCRαβ/CD19-depleted 9/10 MUD-PBSC (single C-antigen HLA mismatch)	Busulfan (AUC: 85 mg/L*h) Fludarabine 150 mg/m^2^ Alemtuzumab 0.6 mg/kg	CsA/MMF	-	3	100%	100%	Survival	None	Remission of colitis

P1 had recurrent otitis and lymphadenitis, and an episode of pneumonia. Pre-transplant %DHR + was 25%. She developed colitis, with histological features consistent with CGD, along with polyarthralgia, discoid lupus, photosensitivity, and weakly positive antinuclear antibodies (1:160), requiring treatment with mycophenolate mofetil (MMF), corticosteroids, and hydroxychloroquine. She underwent HSCT aged 15 years. Post-HSCT, she developed human herpes virus-6 viraemia treated with ganciclovir, an inflammatory pneumonitis and LRTI 5 months post-HSCT, with *Enterobacter* isolated, and autoimmune hemolytic anemia (AIHA) successfully treated with intravenous immunoglobulin (IVIG) and sirolimus. Despite undergoing menarche and having regular menstrual periods prior to HSCT, post-HSCT P1 developed secondary amenorrhea with reduced estradiol (55 pmol/L) and elevated luteinizing hormone (23.1 IU/L) and follicle-stimulating hormone (75.9 IU/L), consistent with ovarian failure. Four years post-HSCT, she remains on hormone replacement therapy with estrogen, but maintains a good level of physical function, has 100% DHR + , and no further infectious or inflammatory phenomena.

P2 presented with a *Staphylococcus aureus* hepatic abscess aged 16 years and recurrent folliculitis causing significant absence from school. Pre-HSCT %DHR + was 10% and genetic analysis identified a large heterozygous deletion (Xp.21.1p.11.4) encompassing *CYBB* along with *OTC* (ornithine transcarbamylase, implicated in hyperammonaemia [24]), *RPGR* (retinitis pigmentosa GTPase regulator, implicated in retinitis pigmentosa [25]), *XK* (X-linked Kx blood group antigen, Kell and VPS13A binding protein, implicated in development of McLeod syndrome [26]), and *TSPAN7* (tetraspanin-7, implicated in X-linked intellectual disability [27]). She had no evidence of neuroacanthocytosis or ornithine transcarbamylase (OTC) deficiency, with a normal blood film and serum ammonia levels. Ophthalmology examination and cranial magnetic resonance imaging were normal. She was well and free from infectious and inflammatory symptoms on admission to the transplant unit for a MUD-PBSC graft aged 18 years, with fludarabine, treosulfan, thiotepa conditioning, and alemtuzumab serotherapy (Table 2). However, the patient rapidly deteriorated with neurological disturbance, hypotension, and anuric renal failure two days prior to stem cell infusion. She was transferred to the pediatric intensive care for cardiovascular and respiratory support and renal replacement therapy and received her stem cell infusion while ventilated. She was treated empirically with broad-spectrum antibiotics and antifungals. There were no positive cultures from urine, endotracheal secretions, or blood but computed tomography demonstrated neutropenic enterocolitis. Due to a sustained inflammatory picture coinciding with neutrophil engraftment, raised C-reactive protein, and high serum IL-6, immunosuppression with methylprednisolone and subsequently tocilizumab was trialed with no change in clinical status. Repeated electroencephalography and magnetic resonance imaging of the patient’s brain showed leukoencephalopathy. Despite significant inotropic support, ventilation, and renal replacement therapy, and engraftment of donor neutrophils with 100% DHR + , she died at D + 30 from multiorgan failure. Post-mortem examination found toxic leukoencephalopathy, presumed from fludarabine or thiotepa used as part of conditioning. Measurement of serum treosulfan, thiotepa, or fludarabine levels was not available.

P3 was previously reported by Hauck et al. [16]. Briefly, she presented with severe colitis requiring multiple immunomodulatory therapies including infliximab, certolizumab, and methotrexate. Her %DHR + was 30%. A heterozygous c.925G > A mutation in *CYBB* was found, causing a substitution of glutamine to lysine at position 309. She underwent HSCT aged 20. She received 14 days of prednisolone prior to chemotherapy induction. Aside from grade 1 acute cutaneous graft-versus-host disease (GvHD) requiring topical therapy, she had an uneventful transplant course and 8 years post-HSCT has 100% donor chimerism with a restored oxidative burst and has sustained clinical and histological remission from her colitis. Post-HSCT, she is nulligravid with an irregular menstrual cycle but normal endogenous estrogen, luteinizing hormone, and follicle-stimulating hormone levels.

P4, previously reported by Trevisan et al. [23], had a long history of colitis initially diagnosed as Crohn’s disease, discoid lupus with Raynaud’s phenomenon, and hypothyroidism. She presented aged 49 with pulmonary *Nocardia* leading to lung resection and a period on extracorporeal life support. Pre-HSCT %DHR + was 6.7% and a c.1662dupT mutation was identified in *CYBB*. She was post-menopausal prior to conditioning. She underwent HSCT aged 56 years, with her autoimmunity relatively controlled on physiological dose corticosteroids pre-conditioning. She developed grade 1 cutaneous GvHD requiring topical therapy and had an episode of *Klebsiella* bacteraemia while aplastic. She was last followed at 3 years post-HSCT, where myeloid chimerism and %DHR + were 60%. Post-HSCT she is free of infection, inflammatory, and autoimmune disease with the exception of pre-existing hypothyroidism.

P5 underwent HSCT aged 22 years, indicated by recurrent infections and complicated colitis with labial and vaginal fistula formation refractory to intensive immunosuppression including azathioprine, corticosteroids, and vedolizumab. At the time of transplant, she had active colitis and was on tapered corticosteroids. She received a parental haploidentical HSCT but developed grade 3 gastrointestinal and liver GvHD which progressed despite intensification of immunosuppression, followed by respiratory adenovirus infection leading to acute respiratory distress syndrome. Despite cidofovir and intensive organ support, and full donor chimerism at D + 100, she died 4 months post-HSCT from multi-organ failure secondary to adenovirus infection.

P6 presented as a child with a significant pneumonia with no positive microbiology and a cutaneous abscess isolating *Burkholderia gladioli*, prompting testing for CGD. Her DHR + aged 6 years was 15%. She was also hypogammaglobulinaemic (IgA: < 0.1 g/L, IgG: 3.63 g/L, IgM: < 0.04 g/L). Lymphocyte subsets showed normal total B-lymphocyte counts (CD19 + : 826 cells/microlitre) but low memory B-lymphocyte populations (IgD-IgM-CD27 + : 0.1%, IgD + IgM + CD27 + : 1.8%, CD38 +  + CD27 + CD20-: 0.1%). Her T-lymphocyte compartment was normal. She underwent next-generation sequencing which identified a heterozygous c.1151 + 2 T > C mutation in *CYBB*, but no other variant to explain her humoral immunodeficiency. She had a discoid rash but no other autoimmune or inflammatory manifestations and underwent HLA-identical sibling HSCT due to her infection history and poor phagocyte function. Post-HSCT, she developed cytomegalovirus (CMV) viraemia, successfully treated with ganciclovir but at 2 years after transplant, she has 100% myeloid and CD19 + chimerism, is off IVIG with good antibody production to vaccine antigens, and has had no infective or inflammatory sequelae.

The final patient, P7, was diagnosed postnatally due to her brother having XL-CGD with colitis. By age 7 months, she had developed colitis with hematochezia and a raised fecal calprotectin (200 μg/g) and was hospitalized for bilateral pneumonia. Due to her brother’s course, unfavorable lyonization with initial DHR + of 1.2%, and her early presentation, she underwent a 9/10 (single C-antigen HLA mismatch) unrelated donor HSCT following TCRαβ/CD19 depletion of the graft. Her HSCT course was uncomplicated and 3 years post-HSCT, she is in remission of colitis with no further infection or inflammatory symptoms. Her latest DHR + is 100%.

## Discussion

In this report, we contacted international alloHSCT centers to collate a series of patients who have undergone alloHSCT indicated by severely symptomatic XL-CGD carrier status. We summarize the transplant experience for this cohort, who represent severe XL-CGD disease despite being carriers.

Similar to patients with classical CGD, a number of XL-CGD carriers suffer from increased mortality, significant symptom burden, and reduced quality of life, for which therapeutic strategies are limited by the impact of long-term immunosuppression on infection risk [12–15, 28]. This is in contrast with existing data on female carriers of some other X-linked inborn errors of immunity such as IL2RG-deficient severe combined immunodeficiency and CD40L deficiency, who appear to be asymptomatic [29]. While female carriers of *CD40L* mutations may be considered as donors for their affected family members, female carriers of XL-CGD should not be used as donors for patients with XL-CGD due to their propensity to develop autoimmunity and the risk of poor phagocyte function from mixed donor chimerism compounded with the dynamic nature of %DHR + over time [4, 29]. For patients with CGD, alloHSCT provides excellent survival and remission of infection susceptibility and inflammatory colitis, particularly for patients with matched sibling donors and those transplanted at a young age regardless of genotype [17, 21, 22], while autologous gene therapy using lentiviral vectors is currently under investigation for XL-CGD and would be a potential therapeutic option for symptomatic XL-CGD carriers [30, 31]. The excellent results in classic CGD may justify use of alloHSCT for symptomatic carriers with impaired %DHR + , particularly given existing data associating percentage of functioning neutrophils with mortality in CGD [32], and disease activity and quality of life [12].

Our patient group demonstrates significant clinical heterogeneity in terms of age and clinical manifestation. Six patients had recurrent infection, five had colitis, and three had lupus-like inflammatory disease, including discoid rash, photosensitivity, and Raynaud’s phenomenon. Notable infective manifestations included bacteria typically associated with CGD, such as *Staphylococcus aureus*, *Nocardia*, and *Burkholderia*; interestingly, despite low %DHR + and significant immunosuppression, no fungal or mycobacterial disease was seen, contrasting with typical CGD. This may relate to sufficient residual phagocyte activity protecting patients from invasive fungal or mycobacterial disease, as low carrier %DHR + predicts infection risk [15, 28]. The majority of patients (five of seven) were diagnosed following a CGD-like clinical manifestation, rather than by family history.

The largest series to describe outcome of HSCT in classical CGD [17] demonstrated decreased survival in patients with active colitis on univariate analysis, in contrast to a previous report showing no significant difference in events such as death and GvHD [28]. This small series cannot provide evidence on the impact of active inflammation at HSCT on outcome: however, it follows that the attendant risks of active colitis at HSCT in classical CGD, namely poor nutrition, epithelial disruption and bacterial translocation, and side effects of immunosuppression, would also likely hold for symptomatic XL-CGD carriers. It is therefore advised that inflammatory manifestations are well-controlled prior to stem cell infusion, though this must be balanced against the infective risks of immunosuppression, and the impact of delaying curative treatment on development of potentially irreversible organ damage.

The choice of conditioning regimen for classical CGD HSCT has previously emphasized myeloablation, in order to reduce the risk of graft failure [18, 33]. However, reduced-intensity approaches incorporating pharmacokinetically guided busulfan, fludarabine, and serotherapy with either alemtuzumab or anti-thymocyte globulin have been reported to lead to excellent overall and event-free survival with graft failure occurring in only 3/56 (5%) patients [22]. A subsequent large European study found no significant impact of conditioning regimen or intensity on overall or event-free survival, and recommended that reduced toxicity busulfan- or treosulfan-based approaches are favored for CGD [17]. The reduced-toxicity approach is recapitulated in the recent update of the EBMT/ESID IEWP guidance on HSCT for inborn errors of immunity [34]. Reducing risk of graft failure and GvHD must be balanced against risk of short- and long-term effects, such as impact on fertility, lung function, and development of metabolic complications. In this series, the five patients receiving busulfan had access to pharmacokinetic-guided dosing with the intention of reducing overall toxicity, and graft failure was not seen. One patient developed ovarian failure requiring estrogen despite receiving a treosulfan-based regimen, which typically impairs ovarian reserve to a lesser extent than myeloablative approaches [35]. This demonstrates the important of appropriate pre-transplant counseling and consideration of fertility preservation.

Three patients developed transplant-related complications, leading to death in two. P1 developed significant autoimmune hemolytic anemia post-HSCT, requiring treatment with high-dose IVIG and sirolimus. This represents an increasingly recognized transplant-related morbidity, with alemtuzumab conditioning identified as a risk factor for its development [36, 37]. The patient maintained high levels of donor chimerism throughout her post-HSCT course and so this was felt to be a de novo event rather than relapse of the autoimmune manifestations seen in XL-CGD carriers. Transplant-related mortality was seen in two patients, occurring in both after sustained engraftment of donor neutrophils and restoration of oxidative function; one patient died due to multiorgan failure and toxic leukoencephalopathy related to conditioning, while the second developed disseminated adenoviral infection following grade III acute GvHD after haploidentical HSCT with PTCy chemoprophylaxis. Toxic leukoencephalopathy has been described rarely in association with fludarabine and thiotepa, with none of the described risk factors (older adults, pre-existing renal disease, previous chemotherapy) being present in this patient [38, 39]. A large retrospective series found a significant survival difference in patients receiving HLA-mismatched grafts (79.5% vs 89.4% for matched family donors), though these data were comprised of a heterogeneous population with respect to conditioning, serotherapy, and graft manipulation [17]. The protocol used for P5, who underwent haploidentical alloHSCT, was based on a non-myeloablative no-serotherapy PTCy/sirolimus regimen from Johns Hopkins [40], but with addition of busulfan in place of irradiation. This protocol has subsequently closed in the setting of haploidentical CGD HSCT following high rates of grade III–IV or steroid-refractory acute GvHD associated with mortality in 2/7 patients in a study by Parta et al. [41], who noted that severe GvHD also associated with active pre-HSCT colitis. While impaired absorption of sirolimus from intestinal inflammation may plausibly contribute to the risk of GvHD in this cohort, it is likely that lack of serotherapy was the main driver of GvHD. A revised protocol including alemtuzumab as serotherapy and without fludarabine is now in use for haploidentical CGD HSCT at this center, without any cases of GvHD in transplanted patients. Furthermore, the protocol used in P4 for matched related or unrelated donor HSCT with PTCy/sirolimus in CGD has also yielded excellent results with respect to GvHD (4/40 cases had GvHD, all grade I–II, and responsive to corticosteroids; manuscript in preparation). Further developments to in vivo and ex vivo T-lymphocyte depletion may further improve outcomes for those patients where a matched donor is unavailable. It is likely that choice of regimen for symptomatic XL-CGD carriers will reflect donor source and developments in conditioning choice for classical CGD.

Given the excellent results in classical CGD, it is unsurprising that alloHSCT in symptomatic XL-CGD carriers successfully restored phagocyte function in 7/7 patients, including those who died of transplant-related complications. The %DHR + test estimates the percentage of neutrophils producing the oxidative burst in response to susceptible stimulus. Patients with classic CGD show virtually no neutrophil oxidative burst activity; most symptomatic XL-CGD carriers show impaired but not completely absent oxidative burst with a dual population of neutrophils, whereas patients expressing wild-type alleles of CGD-associated genes usually demonstrate normal values [12]. All our patients had low pre-treatment %DHR + and demonstrated significant post-transplant restoration in neutrophil function, with six achieving values of 98–100%, and clinical remission in all survivors including the patient with a mixed population of 60% DHR + neutrophils, although longer follow-up is required to determine the risk of developing CGD-associated symptoms. While %DHR + may predict risk of infection in XL-CGD carriers, with < 20% activity associated with severe infection [4], the link between %DHR + activity and risk of autoimmunity is less clear [14]. Autoimmunity in XL-CGD carriers with relatively high %DHR + , such as 60–70%, suggests that the presence of dysfunctional phagocytes may be sufficient to generate abnormal immune reactions [4], supporting the theory that defective efferocytosis is implicated in development of autoinflammation [5, 6, 9]. Indeed, hypomorphic variants in NADPH oxidase genes associate with inflammatory and autoimmune diseases in polygenic studies [42]. Remission of inflammation following restoration of NADPH activity post-HSCT also lends credence to this theory, as we can postulate that efferocytosis will also be restored in these patients.

It is reassuring that patients in this series with inflammatory or lupus-like disease all experienced remission, and no longer require immunosuppression; the four surviving patients with pre-HSCT colitis are all in clinical remission, including the patient previously reported by Hauck et al. However, the sustained remission of symptoms 3 years post-HSCT in the context of mixed myeloid chimerism and 60% DHR + in P4 is surprising. Whether this degree of mixed chimerism will impart a risk of recrudescence of inflammatory symptoms remains to be seen, and will require ongoing surveillance and follow-up. Further characterization of the pathways causing autoimmunity in CGD and XL-CGD carriers may shed light on what degree of donor chimerism is sufficient to remit inflammatory manifestations of this disease. Reduced quality of life (QOL) is a significant feature of inborn errors of immunity including CGD, linked to health burden, restrictions to socio-occupational life, depression, and anxiety [43]. Children are at risk of emotional and psychosocial difficulties [44], missed educational opportunity, and poor growth and development from inflammatory bowel disease. Carriers may experience specific challenges including “genetic guilt” and potentially caring for a child with CGD [12], in addition to the impact of their own physical symptoms. Our data did not explore the impact on QoL for our patients. However, previous data highlight joint symptoms, respiratory illness, colitis, and %DHR + as determinants of poor QOL in symptomatic XL-CGD carriers [12]. The clear resolution of these manifestations by HSCT and absence of chronic GvHD in all survivors suggests that in symptomatic XL-CGD carriers as in classical CGD, HSCT would improve patient-reported measures of QOL [43]. Clearly, further detailed exploration of QOL will aid risk–benefit analysis of treatment strategies for patients.

While the encouraging data for classical CGD support consideration of HSCT in most patients, particularly if transplant is available at a young age prior to accumulation of organ damage, selection of appropriate symptomatic XL-CGD carriers must be individualized to each patient. This is important particularly given the risk inherent in transplant, demonstrated by the deaths of two patients from HSCT-related complications. Symptomatic XL-CGD carriers may present at an older age than classical CGD due to the association between increasing age and skewed X-inactivation, highlighted by P4 in this report; this may further enhance the risks associated with HSCT due to accumulation of end-organ complications. A prospective study by the Inborn Errors Working Party of the European Society for Blood and Marrow Transplantation demonstrated feasibility of reduced-intensity conditioning for HLA-matched alloHSCT in CGD, with event-free survival of 22/24 (92%) adolescent and adult patients [22], and a recent multicentric study of HSCT in adults with IEI was also encouraging, particularly for patients with CGD [45]. This re-emphasizes the importance of serially monitoring %DHR + in all XL-CGD carriers, regardless of symptoms and considering antimicrobial prophylaxis once %DHR + falls < 20%. This is particularly important for patients who are immunosuppressed for treatment of autoimmune manifestations. The wide range of pre-HSCT %DHR + in this cohort (1.2–30%) suggests decisions to offer HSCT should consider the patient’s phenotype and comorbidities, rather than a laboratory cut-off. The small sample size of this study does not allow us to explore whether diagnosis by family history confers a benefit over patients who are diagnosed only after a CGD-like manifestation.

This study is limited by the small sample size reflecting the severe end of the symptomatic XL-CGD carrier symptom spectrum, and the heterogeneous nature of their pre-HSCT clinical status, age, and transplant characteristics. Nonetheless, median follow-up of 3 years in survivors provides reassurance that restoration of oxidative activity and remission of inflammatory symptoms may be maintained past the initial post-transplant phase.

In conclusion, we describe seven patients who have undergone allogeneic HSCT for severe symptomatic XL-CGD carrier status with impaired %DHR + , demonstrating that restoration of neutrophil oxidative burst by engraftment of donor myeloid cells may cure the disease and reverse inflammatory and infective manifestations.

## Data Availability

The data used in this study are not publicly available but may be available from the authors on reasonable request.
